# Influence of prey concentration, light intensity, and temperature on the growth and ingestion of the mixotrophic dinoflagellate *Pyrophacus horologium*, a predator of the harmful species *Heterocapsa niei*

**DOI:** 10.1111/jpy.70150

**Published:** 2026-03-16

**Authors:** Min‐jun Seong, Kun‐woo Yun, Hwa‐seong Son, Seung‐min Lee, Mu‐chan Kim

**Affiliations:** ^1^ Department of Marine Environmental Engineering Gyeongsang National University Tongyeong Republic of Korea; ^2^ Present address: Division of Fisheries Life Sciences Pukyong National University Busan Republic of Korea

**Keywords:** dinoflagellate, harmful algal, *Heterocapsa niei*, ingestion, irradiance, *Pyrophacus horologium*, temperature

## Abstract

*Pyrophacus horologium* belongs to the family Pyrocystaceae and was recently identified as a mixotrophic dinoflagellate capable of feeding on other dinoflagellates. In this study, the growth and feeding responses of *P. horologium* were investigated under various environmental conditions by providing *Heterocapsa niei*, its preferred prey. The mixotrophic growth rate was measured in a feed concentration range of approximately 350 to 35,000 cells · mL^−1^, reaching saturation at approximately 8343 cells · mL^−1^. In the temperature experiment conducted under mixotrophic conditions (10–35°C at 90 μmol photons · m^−2^ · s^−1^), positive growth was observed between 15 and 32°C, with the maximum mixotrophic growth rate of 0.48 · day^−1^ recorded at 30°C (at 90 μmol photons · m^−2^ · s^−1^). In the light intensity experiment conducted under mixotrophic conditions (0–348 μmol photons · m^−2^ · s^−1^ at 30°C), positive growth was observed between 24 and 170 μmol photons · m^−2^ · s^−1^, with the maximum mixotrophic growth rate of 0.38 · day^−1^ at 120 μmol photons · m^−2^ · s^−1^ (at 30°C). The maximum ingestion rates observed were 30.2 cells · predator^−1^ · day^−1^ (at 28°C and 90 μmol photons · m^−2^ · s^−1^) and 28.5 cells · predator^−1^ · day^−1^ (at 30°C and 120 μmol photons · m^−2^ · s^−1^). The result indicate that growth rates were significantly affected by temperature and light intensity. Collectively, these results provide insights into the environmental adaptability of *P. horologium* and its potential ecological implications, enhancing our understanding of mixotrophic dinoflagellates that graze on the harmful species *H. niei*.

**Abbreviations**
ANOVA: analysis of variance
HABs: harmful algal blooms
HSD: honestly significant difference
IOGZ: irradiance‐based optimal growth zone
ISZ: irradiance‐based survival zone
MANOVA: multivariate analysis of variance
PAR: photosynthetically active radiation
TOGZ: temperature‐based optimal growth zone
TSZ: temperature‐based survival zone

## INTRODUCTION

Dinoflagellates are ubiquitous microalgae that exist in abundance in marine ecosystems and play a variety of ecological roles, including those of primary producers, prey, predators, parasites, and symbionts (Jeon & Park, 2024; Ok & Jeong, 2025; Zhao et al., 2024). The classification of these organisms is determined by their mode of nutrition, which can be autotrophic, heterotrophic, or mixotrophic. Specifically, mixotrophy has gained attention as a survival strategy that maximizes energy acquisition through a combination of photosynthesis and phagotrophy (Johnson, 2015). Furthermore, mixotrophy has been demonstrated to enhance adaptability to environmental changes and contribute to increased growth and survival rates (Jeong et al., 2021).

In recent decades, a considerable number of dinoflagellates previously classified as autotrophic have been observed to exhibit mixotrophic behavior (Bockstahler & Coats, 1993; Jacobson & Anderson, 1996). In addition, some species have been reported to contribute not only to red tide formation but also to the control of harmful species (Menden‐Deuer & Montalbano, 2015; Park et al., 2013). For example, *Alexandrium pohangense* and *Fragilidium mexicanum* have been suggested to possess the potential to control harmful dinoflagellates by feeding on *Margalefdinium polykrikoides* and *Alexandrium* spp., respectively (Kim & Park, 2017; Lim et al., 2015). However, among the extensive array of dinoflagellate species, only a limited number have been documented to exhibit mixotrophic characteristics (Jang et al., 2017; Lee et al., 2014; Ok et al., 2023; Seong et al., 2025; You, Ok, et al., 2023). In addition, studies on the physiological traits and ecological functions of individual mixotrophic species remain limited (Coyne et al., 2021; Kang et al., 2020; Li et al., 1999; Liu et al., 2021).

*Pyrophacus horologium*, a thecate dinoflagellate, was recently reported as a mixotrophic species capable of completely engulfing and consuming other dinoflagellates through an engulfment feeding mechanism (Seong et al., 2025). *Pyrophacus horologium* is distributed across tropical to temperate marine environments, but it has generally been reported as a species of moderate abundance rather than a dominant taxon (Dale, 1977; Khairy et al., 2014; Nunes et al., 2019; Pednekar et al., 2011). This species has been documented consuming a variety of thecate dinoflagellates, including the harmful species *Heterocapsa niei*, as well as *Alexandrium* spp., *Gonyaulax spinifera*, *Prorocentrum* spp., and *Scrippsiella trochoidea*. These prey species have been documented in natural marine environments as either toxin producers or causative agents of harmful algal blooms (Ichimi et al., 2002; Lu & Goebel, 2001; Rhodes et al., 2006; Yin et al., 2008). Preliminary experiments on *P. horologium* indicated that mixotrophic conditions were more conducive to growth than autotrophic conditions, suggesting that this species derives physiological advantages from prey ingestion. This feeding behavior may also play an ecological role in regulating populations of harmful dinoflagellates, such as *H. niei*.

The growth and ingestion of mixotrophic dinoflagellates are influenced by a variety of environmental factors, including prey availability, as well as temperature, light intensity, and salinity (Jeong et al., 1999; Kim et al., 2004; Parkhill & Cembella, 1999). Specifically, light intensity and temperature have been identified as critical factors that directly influence the physiological activity and viability of cells (Singh & Singh, 2015). Among these factors, light intensity has emerged as a pivotal environmental element that exhibits daily and seasonal variations within marine ecosystems, exerting a direct influence on the life cycle of dinoflagellates (Côté & Platt, 1983). Many dinoflagellates undergo diel vertical migration, performing photosynthesis near the surface during the day and descending to nutrient‐rich deeper layers at night, thereby experiencing a wide range of light conditions (Blasco, 1978; Park et al., 2002). Some species have been observed to survive and continue ingestion for a certain period under dark conditions (Jakobsen et al., 2000; Stoecker et al., 1997), whereas excessively high light intensities have been shown to cause photoinhibition, leading to suppressed growth (Kibler et al., 2012; López‐Rosales et al., 2014).

Temperature is one of the major environmental factors that influences metabolism, cell division, growth, and survival in dinoflagellates (Montagnes et al., 2003; Wang et al., 2022). Generally, the growth rate of dinoflagellates tends to increase with rising temperature; however, exceeding a certain threshold can induce physiological stress, leading to growth inhibition or cell death (Jang et al., 2018; Yamaguchi et al., 2012). Consequently, each species possesses its own optimal temperature and a tolerable thermal range, which exert a profound influence on its spatial distribution and seasonal occurrence patterns (Dela‐Cruz et al., 2008; Laabir et al., 2011). Therefore, a quantitative evaluation of the effects of prey concentration, light intensity, and temperature on the growth and ingestion rates of *Pyrophacus horologium* is necessary.

In this study, the growth and ingestion rates of *Pyrophacus horologium* isolated from Masan Bay, Korea, were measured under a range of prey concentrations (350–35,000 cells · mL^−1^), light intensities (0–348 μmol photons · m^−2^ · s^−1^), and temperature conditions (10–35°C). Furthermore, based on the field‐measured environmental conditions and experimental data from Masan Bay, a modeling approach was applied to evaluate the potential survivability and ecological dominance of *P. horologium* under changing marine environmental conditions. This study utilized quantitative analysis to elucidate the physiological and ecological characteristics of *P. horologium*, emphasizing its potential ecological role as a mixotrophic dinoflagellate that preys on harmful species in marine ecosystems. The study further proposes *P. horologium* as a model organism for studying mixotrophic strategies.

## MATERIALS AND METHODS

### Collection and culture

The *Pyrophacus horologium* strain GNMS23 was isolated from a seawater sample collected from the coastal waters of Masan Bay, Korea, in June 2023, when the surface water temperature and salinity were 23.7 and 30.7°C, respectively (Seong et al., 2025). *Heterocapsa niei*, isolated from the same location in May 2024, was used as the prey organism. The temperature and salinity were documented as 26.1 and 23.0°C, respectively, at the time of isolation. The cultivation of both species occurred under autotrophic conditions using F/2‐Si medium, prepared with sterile natural seawater (Guillard & Ryther, 1962). The cultures were maintained at 22°C in an algal incubator (JSMI‐04CPL, JSR, Korea) under a 14:10 h light:dark photoperiod. Cool white, fluorescent lamps served as the light source, with the light intensity maintained at 90 μmol photons · m^−2^ · s^−1^ during the culture period. The light intensity was measured using a portable light meter (LI‐250A, LI‐COR, Inc., United States). Cell volume was obtained from the dataset reported by Seong et al. (2025; *n* = 50), and cellular carbon content was estimated using the equation provided by Strathmann (1967). Consequently, the cellular carbon content was estimated to be 4.97 ng C · cell^−1^ for *P. horologium* and 0.079 ng C · cell^−1^ for *H. niei*.

### Prey concentration effects

This experiment was designed to quantitatively assess the growth and ingestion rates of *Pyrophacus horologium* in response to varying prey concentrations of *Heterocapsa niei*. The predator and prey cells utilized in the experiment were obtained from 3‐L autotrophic cultures maintained in the exponential growth phase. During the light phase of the light–dark cycle, cells formed a dense layer along the illuminated side of the flasks, and this high‐density cell layer was gently collected using an electronic pipette. The initial cell concentrations were determined using an inverted microscope (ECLIPSE Ts2, Nikon, Tokyo, Japan) and a micropipette. The initial concentrations of *P. horologium* and *H. niei* were established at approximately 366 cells · mL^−1^ and 121,200 cells · mL^−1^, respectively. The experimental design involved the utilization of 63 polycarbonate (PC) bottles and the implementation of seven levels of prey concentration. The experiment involved the replication of mixed cultures of predators and prey, as well as predator and prey controls, on three separate occasions. Each bottle contained 40‐mL of culture medium. To ensure consistent water conditions among treatments, each culture of the predator or prey was filtered through a 0.7‐μm GF/F filter, and the resulting filtrate was added to the corresponding control bottles in the same volume as that used for the mixed cultures. This procedure was applied to equalize the dissolved organic and nutrient background between the experimental and control treatments. Then, 5 mL of the F/2‐Si medium, which was prepared using sterile natural seawater, was added to each bottle. The remaining volume was filled with a sterile solution of seawater. The incubation was conducted in an algal incubator at 22°C under a 14:10 h light:dark photoperiod with a light intensity of 90 μmol photons · m^−2^ · s^−1^. To assess cell density, 1‐mL samples were taken from each bottle at the beginning and after a 2‐day incubation, and both were preserved with Lugol's solution for counting. The final initial cell concentrations utilized in the experiment are enumerated below (in cells · mL^−1^): 6–156/0; 6/350; 12/1183; 27/4358; 40/9933; 73/17,433; 107/25,367; and 152/35,000 (*P. horologium*/*H. niei*).

The specific growth rate (μ, day^−1^) of *P. horologium* was determined by utilizing the following equation:

(1) $$\mu =\frac{\mathrm{Ln}\left(P_{t}/P_{0}\right)}{t}$$

In this equation, *P*_0_ denotes the initial predator cell density, and *P*_t_ represents the predator cell density at time *t* (in days).

The derived growth rate was subsequently implemented as the following Michaelis–Menten equation:

(2) $$\begin{array}{c} \mu =\frac{\mu_{\text{max}}\left(x-x^{\prime }\right)}{K_{\mathrm{GR}}+\left(x-x^{\prime }\right)} \end{array}$$

In this equation, μ_max_ is defined as the maximum specific growth rate (day^−1^), *x* is the concentration of *Heterocapsa niei* (cells · mL^−1^ or ng C · mL^−1^), *x′* denotes the threshold prey concentration at which the growth rate becomes zero, and *K*_GR_ is the prey concentration required to sustain half of μ_max_.

The ingestion rate (*I*) and clearance rate were calculated using equations from Frost (1972) and Heinbokel (1978), respectively. The derived ingestion rate was subsequently implemented as the following Michaelis–Menten equation:

(3) $$\begin{array}{c} \mathrm{IR}=\frac{I_{\max}\left(x\right)}{K_{\mathrm{IR}}+\left(x\right)} \end{array}$$

In this equation, *I*_max_ denotes the maximum ingestion rate (cells · predator^−1^ · day^−1^ or ng C · predator^−1^ · day^−1^), *x* represents the concentration of *Heterocapsa niei* (cells · mL^−1^ or ng C · mL^−1^), and *K*_IR_ represents the prey concentration required to maintain half of *I*_max_. All analyses of growth and ingestion rates were conducted using R version 4.4.1, and graphical visualizations were generated using the same software.

### Temperature effects

In order to assess the impact of temperature fluctuations on the mixotrophic growth and ingestion rates of *Pyrophacus horologium*, a set of two experiments (1 and 2) were conducted (Table 1). The outcomes of the prey concentration experiment were reflected in the temperature experiment, which established the prey concentration above saturation to ensure that *P. horologium* did not experience growth inhibition. In experiment 1, the autotrophic and mixotrophic growth rates, as well as ingestion rates, of *P. horologium* were measured under six different temperature conditions. In this experimental setting, the predators and prey were harvested from autotrophic, densely cultivated cells in 3‐L flasks. The initial cell concentrations were approximately 553 cells · mL^−1^ for *P. horologium* and 74,100 cells · mL^−1^ for *Heterocapsa niei*. The acclimation period to temperature was set to 8 days, based on the autotrophic growth rate of *P. horologium* (0.25 · day^−1^), allowing for a minimum of two cell divisions, as previously described by Ok et al. (2019). During this period, predator cells were subjected to incubation in 250‐mL PC bottles within algal incubators, which were set to the target temperatures (Figure S1). Subsequent to acclimation, the experimental design involved the utilization of 54 PC bottles and the implementation of six levels of temperature. The experiment involved the replication of mixed cultures of predators and prey, as well as predator and prey controls, on three separate occasions. All experimental conditions, with the exception of temperature, were maintained identically to those utilized in the prey concentration experiment. During the incubation period, growth inhibition was observed at temperatures of 10, 15, and 35°C. Consequently, the 8‐day acclimation process was reinitiated at these temperatures, and the experiment was repeated (Figure S1). The initial cell concentrations employed in the experiment are enumerated in Table 1.

**TABLE 1:** Initial concentrations and experimental design for *Pyrophacus horologium* and *Heterocapsa niei* (cells · mL^−1^).

Expt.	Fixed condition	Condition	Initial predator concentration	Initial prey concentration
1 (Tm)	90 μmol photons · m^−2^ · s^−1^	10, 15, 20, 25, 30, 35 (°C)	114, 123, 140, 124, 119, 121	26,733, 26,100, 23,800, 24,200, 23,200, 20,567
2 (Tm)	90 μmol photons · m^−2^ · s^−1^	28, 32 (°C)	127, 145	26,067, 19,933
3 (Li)	30°C	0, 6, 15, 24, 56, 120, 242, 348 (μmol photons · m^−2^ · s^−1^)	97, 112, 123, 111, 154, 140, 104, 86	23,333, 21,400, 23,600, 27,100, 23,467, 27,467
4 (Li)	30°C	90, 170 (μmol photons · m^−2^ · s^−1^)	148, 110	24,333, 23,267

Abbreviations: Expt., Experiment; Li, light intensity; Tm, Temperature.

Experiment 2 was conducted to achieve more precise measurements regarding the optimal growth temperature of *Pyrophacus horologium*. In experiment 2, a narrower temperature range centered around the highest growth rate observed at 30°C (at 90 μmol photons · m^−2^ · s^−1^) in experiment 1 was applied. In experiment 2, the predator and prey cells were harvested from autotrophic, dense cultures maintained in 3‐L flasks. The initial cell concentrations were determined to be approximately 810 cells · mL^−1^ for *P. horologium* and 92,000 cells · mL^−1^ for *Hetercapsa niei*. The experimental conditions and duration were consistent with those employed in experiment 1. The growth rate was calculated using Equation (1), and ingestion rates were estimated based on the methods of Frost (1972) and Heinbokel (1978).

In addition, in order to compare with field conditions, sea surface temperature data from the Masan Bay station, from where *Pyrophacus horologium* was originally isolated, were obtained from the Marine Environment Information System (MEIS) for the period from January 2023 to February 2025 (KOEM, 2025). The temperature data were processed as daily means, and in conjunction with the experimentally determined temperature‐dependent growth rates, a spline function was applied to evaluate the thermal range for potential survival and high growth of the species.

### Light effects

The light intensity experiments were conducted at a prey concentration that reflected the saturating prey concentration determined in the prey concentration experiment. The experiment was also carried out at 30°C, which yielded the highest growth rate in the temperature experiment (Table 1). Experiments (3 and 4) were conducted to evaluate the growth and ingestion rates of *Pyrophacus horologium* under autotrophic and mixotrophic conditions. In these experiments, the organism was fed on *Hetercapsa niei* across a range of light intensities. The predator and prey cells utilized in experiment 3 were harvested from dense autotrophic cultures maintained in 3‐L flasks. The initial cell concentrations were determined to be approximately 1226 cells · mL^−1^ for *P. horologium* and 62,000 cells · mL^−1^ for *H. niei*. The desired irradiance levels were adjusted by modifying the distance between the culture bottles and the LED light source (FS‐050 W, 6500 K; Barom Inc., Seoul, Korea). The dark condition (0 μmol photons · m^−2^ · s^−1^) was achieved by employing a complete blockage of light using aluminum foil wrapped around the bottles. The cells were acclimated to the target light conditions for 8 days in 250‐mL PC bottles placed in algal incubators set to the corresponding irradiance levels (Figure S2). Subsequent to acclimation, the experimental design involved the utilization of 72 PC bottles and the implementation of eight levels of irradiance. The experiment involved the replication of mixed cultures of predators and prey, as well as predator and prey controls, on three separate occasions. All experimental conditions and incubation durations, with the exception of irradiance and temperature (30°C), were identical to those utilized in the temperature experiments. During the incubation period, growth inhibition was observed at irradiances of 0, 6, 15, 242, and 348 μmol photons · m^−2^ · s^−1^. Consequently, the 8‐day acclimation process was reinitiated at these irradiances, and the experiment was repeated (Figure S2). The initial cell concentrations employed in the experiment are enumerated in Table 1.

Experiment 4 was conducted to achieve more precise measurements regarding the optimal growth irradiance of *Pyrophacus horologium*. In experiment 4, a narrower irradiance range centered around the highest growth rate observed at 120 μmol photons · m^−2^ · s^−1^ (at 30°C) in experiment 3 was applied. In the present experiment, the predator and prey cells were harvested from autotrophic, dense cultures maintained in 3‐L flasks. The initial cell concentrations were determined to be approximately 1440 cells · mL^−1^ for *P. horologium* and 92,400 cells · mL^−1^ for *Heterocapsa niei*. The experimental conditions and duration were consistent with those employed in experiment 3. The growth rate was calculated using Equation (1), and ingestion rates were estimated based on the methods of Frost (1972) and Heinbokel (1978).

In addition, environmental data from five monitoring stations in proximity to the original isolation site of *Pyrophacus horologium* were obtained from the MEIS. The dataset for each station included the average depth, secchi disk transparency, and latitude and longitude recorded in August 2023. Subsequently, the values were subjected to averaging for each station, and these values were then employed in the ensuing analysis. To analyze irradiance environments, representative photosynthetically active radiation (PAR) values were determined by averaging the top 10% of daily PAR measurements recorded at each station. The PAR data under clear‐sky conditions were obtained from the National Aeronautics and Space Administration's Prediction of Worldwide Energy Resources (POWER version 2.4.14) database (NASA, 2025), and the light attenuation coefficient was estimated using the equation proposed by Poole and Atkins (1929). Subsequently, the mixotrophic growth rates measured at each light intensity level were analyzed using a spline function, in conjunction with field‐based data on depth, light conditions, and swimming speed.

### Statistical analysis

The statistical analyses were conducted using R software version 4.4.1 (R Core Team, 2024). Univariate analysis was performed to evaluate the effects of water temperature and light intensity on the growth and ingestion rates of *Pyrophacus horologium* under autotrophic and mixotrophic conditions. The normality assumption was verified using the Shapiro–Wilk's W test, and the homogeneity of variance assumption was verified using the Levene's test. When both conditions were met, one‐way analysis of variance (ANOVA) was applied, and Tukey's honestly significant difference (HSD) test was used for post hoc comparisons.

Multivariate analysis of variance (MANOVA) was performed to evaluate the complex response of autotrophic and mixotrophic growth rates to changes in water temperature and light intensity. In addition, normality was assessed through the implementation of the Shapiro–Wilk's W test, homogeneity of variance was evaluated using Box's M test, and significance testing was conducted based on Pillai's trace statistic (Johnson & Field, 1993).

Furthermore, an independent samples *t*‐test was conducted to ascertain whether there was a significant difference in growth rate between autotrophic and mixotrophic under identical environmental conditions and whether the ingestion rate differed significantly from zero. In the course of all statistical analyses, a significance level of *p* < 0.05 was established.

### Swimming speed

The swimming speed of densely cultured *Pyrophacus horologium* (ca. 664 cells · mL^−1^) and *Hetercapsa niei* (ca. 58,200 cells · mL^−1^) was measured by dispensing each culture solution into a 6‐well plate and stabilizing it for 20 min under the same conditions as the culture environment. Subsequently, images were captured for a period of 5 min at 10× magnification using a CMOS camera (E3ISPM20000KPA, AMDSP) mounted on a stereo microscope. The recorded video was captured at a rate of five frames per second and subsequently converted to the AVI format. The videos were then processed using the tracking plugin of ImageJ/Fiji (version 2.16.0), as outlined by Holme et al. (2023). The implementation of track filtering was contingent upon the number of frames and the swimming speed. Tracks with very short frame counts or average speeds that deviated from the overall average by ±2 standard deviations were excluded from the analysis. Finally, the swimming speed of each cell was calculated based on the coordinate change between frames, and the analysis was performed using a total of more than 30 valid track data sets.

## RESULTS

### Prey concentration effects

**FIGURE 1:**
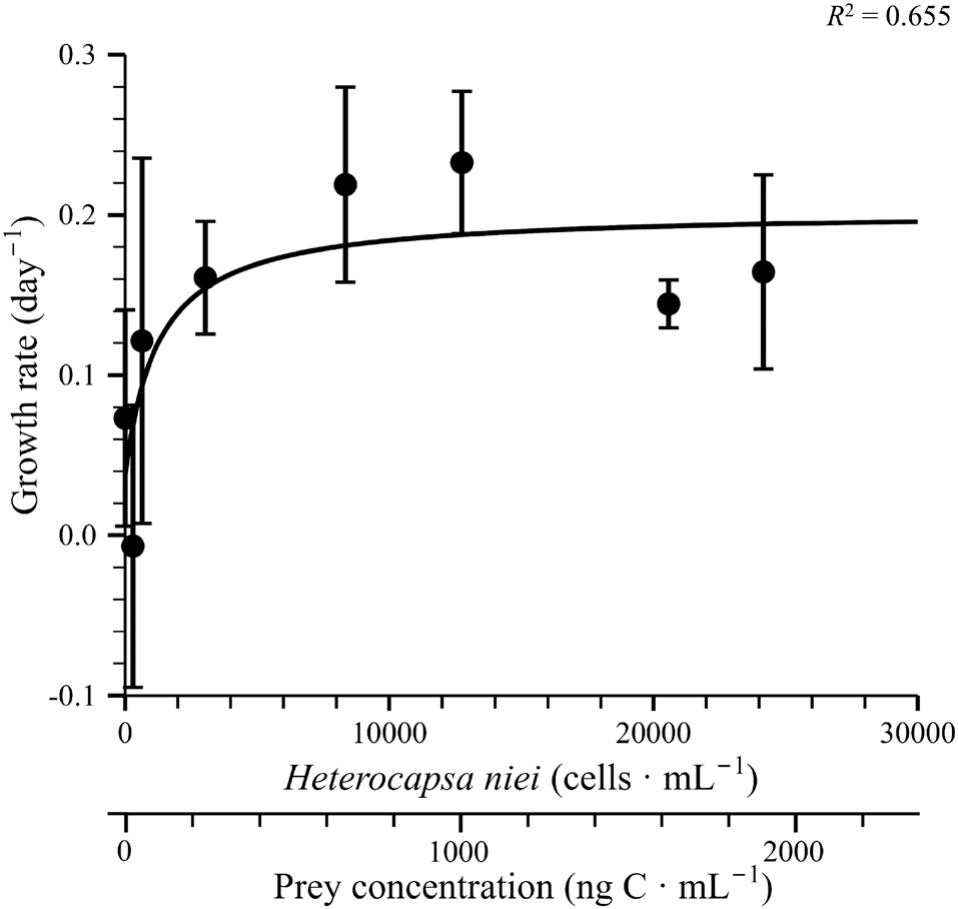
Specific growth rates of *Pyrophacus horologium* under mixotrophic conditions as a function of mean prey concentrations (cells · mL^−1^ and ng C · mL^−1^). Each point represents the mean of three replicates, and vertical error bars indicate ±1 standard error. The regression curve was fitted using the Michaelis–Menten equation [Equation (2)]: Growth rate (GR, day^−1^) = 0.20 [(*x* + 232)/(1023 + (*x* + 232))], *R* ^2^ = 0.66.

The specific growth rate of *Pyrophacus horologium* exhibited a substantial increase in response to elevated average prey concentrations, attaining a maximum at approximately 8343 cells · mL^−1^, or 659 ng C · mL^−1^ (Figure 1). When the maximum growth rate (μ_max_) of *P. horologium* feeding on *Heterocapsa niei* was estimated by fitting the Michaelis–Menten equation Equation (2), the results indicated a maximum growth rate of 0.20 · day^−1^ at 22°C, 90 μmol photons · m^−2^ · s^−1^, and a 14:10 h light:dark cycle. Conversely, in the absence of a prey supply under autotrophic nutritional conditions, the growth rate exhibited a lower value of 0.070 · day^−1^.

**FIGURE 2:**
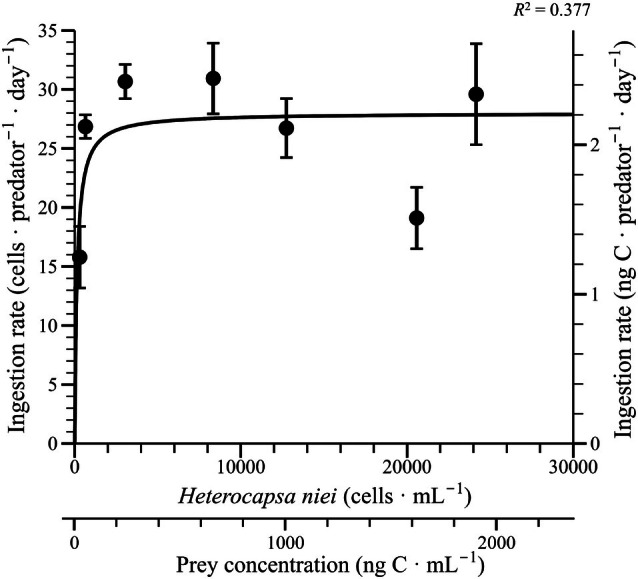
Ingestion rates of *Pyrophacus horologium* under mixotrophic conditions as a function of mean prey concentrations (cells · mL^−1^ and ng C · mL^−1^). Each point represents the mean of three replicates, and vertical error bars indicate ±1 standard error. The regression curve was fitted using the Michaelis–Menten equation [Equation (3)]: Ingestion rate (IR, cells · predator^−1^ · day^−1^) = 28.0 [*x*/(139 + *x*)], *R* ^2^ = 0.38.

The ingestion rate of *Pyrophacus horologium* consuming *Heterocapsa niei* exhibited a marked increase up to an average prey concentration of 642 cells · mL^−1^ (51 ng C · mL^−1^). Consequently, the ingestion rate achieved a state of saturation as the concentration increased (Figure 2). The ingestion rate and clearance rate data obtained directly were fit to the Michaelis–Menten equation Equation (3). The maximum ingestion rate (*I*_max_) of *P. horologium* consuming *H. niei* under the same experimental conditions was estimated to be 28.0 cells · predator^−1^ · day^−1^ (2.21 ng C · predator^−1^ · day^−1^). The maximum clearance rate was calculated to be 2.26 μL · predator^−1^ · h^−1^. Observations made at elevated prey concentrations revealed a slight decline beyond the saturation plateau, both in terms of growth and ingestion.

### Temperature effects

In prey‐only control conditions, *Heterocapsa niei* demonstrated growth within a temperature range of 20–32°C (Figure S3). The lowest growth was observed at 35°C (severe inhibition), while the maximum growth was recorded at 30°C (at 90 μmol photons · m^−2^ · s^−1^).

**FIGURE 3:**
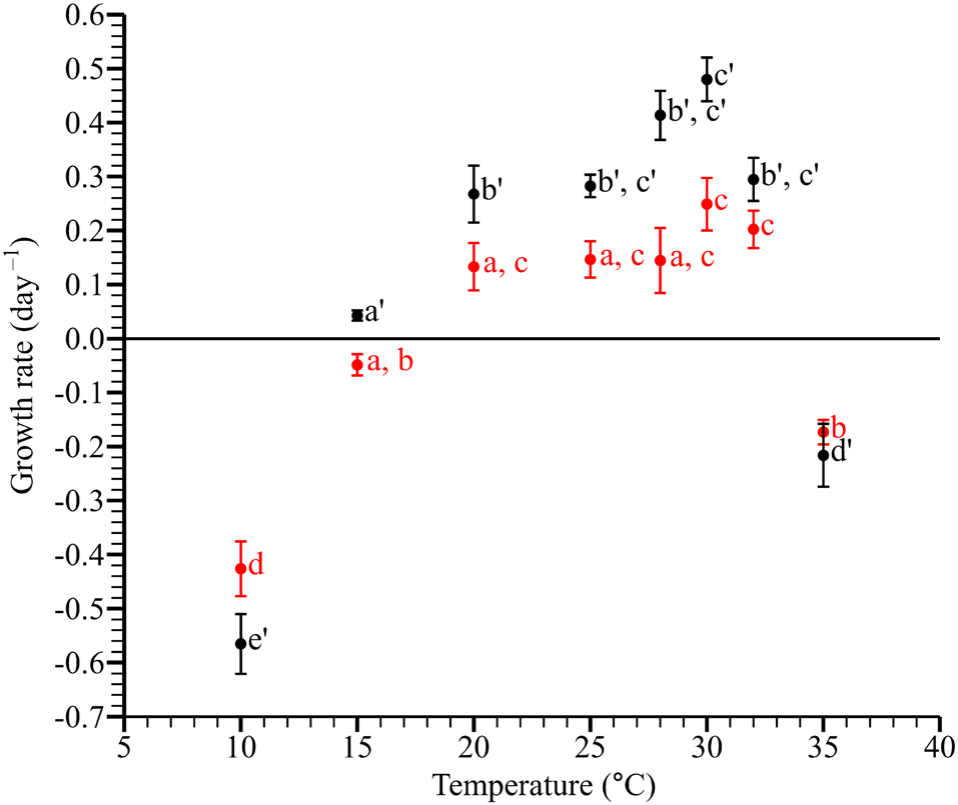
Autotrophic (red markers) and mixotrophic (black markers) growth rates of *Pyrophacus horologium* under different temperature conditions. Each point represents the mean of three replicates, and vertical error bars indicate ±1 standard error. According to Tukey's HSD test, the groupings for autotrophic growth rates were as follows: 10 (d), 15 (a, b), 20 (a, c), 25 (a, c), 28 (a, c), 30 (c), 32 (c) and 35°C (b); and for mixotrophic growth rates: 10 (e'), 15 (a'), 20 (b'), 25 (b', c'), 28 (b', c'), 30 (c'), 32 (b', c') and 35°C (d').

In autotrophic conditions, *Pyrophacus horologium* demonstrated growth within a temperature range of 20–32°C, exhibiting a growth rate of 0.13–0.25 · day^−1^ (Figure 3). The lowest recorded growth rate was observed at 10°C, with a rate of −0.43 · day^−1^, while the maximum growth rate was recorded at 30°C (at 90 μmol photons · m^−2^ · s^−1^). The results of one‐way ANOVA revealed that the autotrophic growth rate differed significantly depending on temperature (*F*_7,16_ = 30.81, *p* < 0.001) and was classified into four temperature groups through Tukey's HSD post hoc test (*p* < 0.05; Figure 3).

In mixotrophic conditions, *Pyrophacus horologium* demonstrated growth within a temperature range of 15–32°C, with growth rates ranging from 0.04–0.48 · day^−1^ (Figure 3). The lowest recorded growth rate was observed at 10°C, with a rate of −0.57 · day^−1^, while the maximum growth rate was recorded at 30°C. The results of one‐way ANOVA revealed that the mixotrophic growth rate differed significantly depending on temperature (*F*_7,16_ = 67.05, *p* < 0.001) and was classified into four temperature groups through Tukey's HSD post hoc test (*p* < 0.05; Figure 3).

The MANOVA revealed a significant effect of temperature on the growth rates under both autotrophic and mixotrophic conditions (Pillai's trace = 1.151, *F*_7,16_ = 3.10, *p* = 0.004). Independent sample *t*‐tests conducted at each temperature condition revealed no significant difference in growth rates (*p* > 0.05) between the two trophic modes at 10 (*t*_4_ = 1.86, *p* = 0.138), 20 (*t*_4_ = −1.96, *p* = 0.124), 32 (*t*_4_ = −1.75, *p* = 0.157), and 35°C (*t*_3_ = 0.689, *p* = 0.547). However, substantial differences were detected at 15 (*t*_3_ = −4.15, *p* = 0.028), 25 (*t*_3_ = −3.43, *p* = 0.035), 28 (*t*_4_ = −3.57, *p* = 0.027), and 30°C (*t*_4_ = −3.65, *p* = 0.023).

**FIGURE 4:**
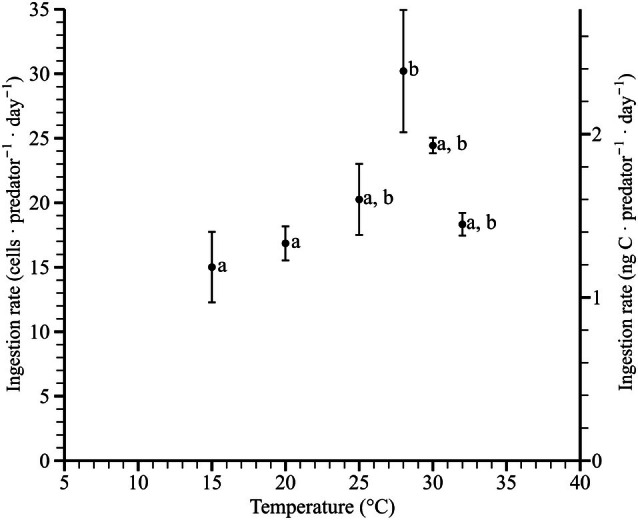
Ingestion rates of *Pyrophacus horologium* under different temperature conditions. The temperatures of 10 and 35°C were excluded due to negative growth rates. Each point represents the mean of three replicates, and vertical error bars indicate ±1 standard error. According to Tukey's HSD test, groupings for ingestion rates were as follows: 15 (a), 20 (a), 25 (a, b), 28 (b), 30 (a, b), and 32°C (a, b).

In mixotrophic conditions, the ingestion rates of *Pyrophacus horologium* were measured across a temperature range of 15–32°C, ranging from 15.0–30.2 cells · predator^−1^ · day^−1^ (Figure 4). The 10 and 35°C conditions, where negative growth rates were observed, were excluded from the ingestion rate analysis. The highest ingestion rate was recorded at 28°C. The results of one‐way ANOVA revealed that the ingestion rate differed significantly depending on temperature (*F*_5,12_ = 4.67, *p* = 0.013) and was classified into two temperature groups through Tukey's HSD post hoc test (*p* < 0.05; Figure 4). One‐tailed *t*‐tests conducted for each temperature condition indicated that ingestion rates were significantly higher than zero at all tested temperatures. The following temperatures were identified as significant: 15 (*t*_2_ = 5.50, *p* = 0.016), 20 (*t*_2_ = 12.8, *p* = 0.003), 25 (*t*_2_ = 7.35, *p* = 0.009), 28 (*t*_2_ = 6.37, *p* = 0.012), 30 (*t*_2_ = 40.2, *p* = 0.001), and 32°C (*t*_2_ = 20.7, *p* = 0.001).

### Light effects

In prey‐only control conditions, *Heterocapsa niei* demonstrated growth within a light intensity range of 24–120 μmol photons · m^−2^ · s^−1^ (Figure S4). The lowest growth was observed in darkness (0 μmol photons · m^−2^ · s^−1^), while the maximum growth was recorded at 120 μmol photons · m^−2^ · s^−1^ (at 30°C).

**FIGURE 5:**
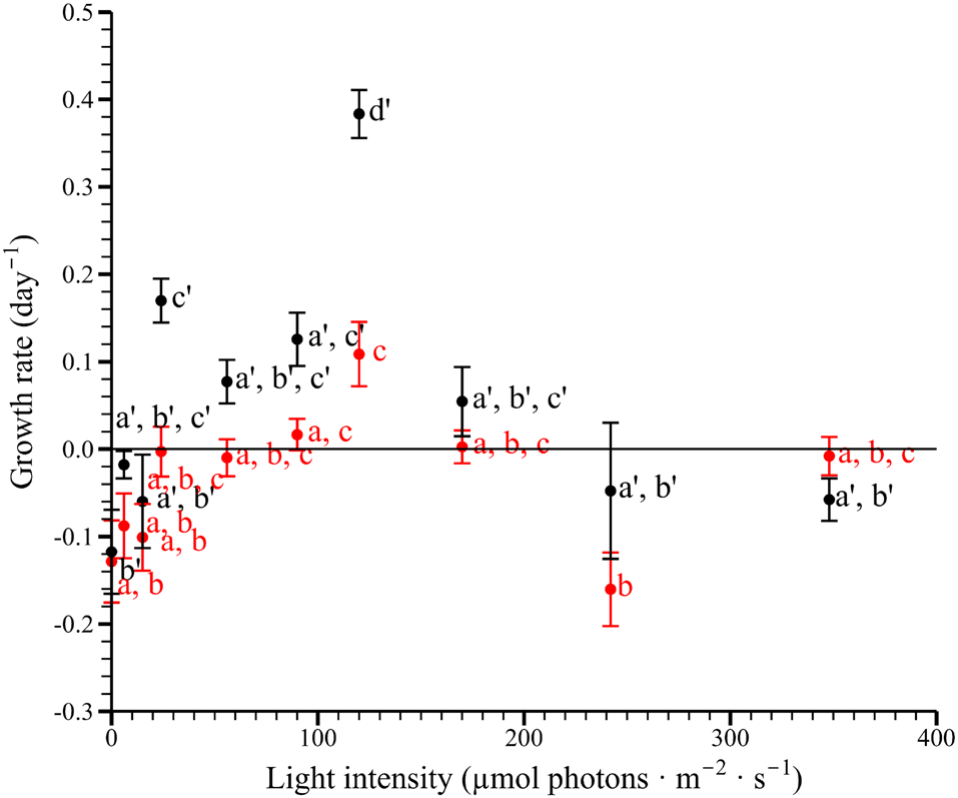
Autotrophic (red markers) and mixotrophic (black markers) growth rates of *Pyrophacus horologium* under different light intensities. Each point represents the mean of three replicates, and vertical error bars indicate ±1 standard error. According to Tukey's HSD test, the groupings for autotrophic growth rates were as follows: 0 (a, b), 6 (a, b), 15 (a, b), 24 (a, b, c), 56 (a, b, c), 90 (a, c), 120 (c), 170 (a, b, c), 242 (b), and 348 μmol photons · m^−2^ · s^−1^ (a, b, c); and for mixotrophic growth rates: 0 (b'), 6 (a', b', c'), 15 (a', b'), 24 (c'), 56 (a', b', c'), 90 (a', c'), 120 (d'), 170 (a', b', c'), 242 (a', b'), and 348 μmol photons · m^−2^ · s^−1^ (a', b').

In autotrophic conditions, *Pyrophacus horologium* demonstrated growth within a light intensity range of 90–170 μmol photons · m^−2^ · s^−1^, exhibiting a growth rate of 0.003–0.109 · day^−1^ (Figure 5). The lowest recorded growth rate was observed at 242 μmol photons · m^−2^ · s^−1^, with a rate of −0.16 · day^−1^, whereas the maximum growth rate was recorded at 120 μmol photons · m^−2^ · s^−1^ (at 30°C). The results of one‐way ANOVA revealed that the autotrophic growth rate differed significantly depending on light intensity (*F*_9,20_ = 6.151, *p* < 0.001) and was classified into three light intensity groups through Tukey's HSD post hoc test (*p* < 0.05; Figure 5).

In mixotrophic conditions, *Pyrophacus horologium* exhibited growth within a light intensity range of 24–170 μmol photons · m^−2^ · s^−1^, with growth rates ranging from 0.05–0.38 · day^−1^ (Figure 5). The lowest recorded growth rate was observed at 0 μmol photons · m^−2^ · s^−1^, with a rate of −0.12 · day^−1^, whereas the maximum growth rate was recorded at 120 μmol photons · m^−2^ · s^−1^. The results of one‐way ANOVA revealed that the mixotrophic growth rate differed significantly depending on light intensity (*F*_9,20_ = 13.29, *p* < 0.001) and was classified into four light intensity groups through Tukey's HSD post hoc test (*p* < 0.05; Figure 5).

The MANOVA revealed a significant effect of light intensity on the growth rates under both autotrophic and mixotrophic conditions (Pillai's trace = 1.253, *F*_9,20_ = 3.72, *p* < 0.001). Independent sample *t*‐tests conducted at each light condition revealed no significant difference (*p* > 0.05) in growth rates between the two trophic modes at 0 μmol photons · m^−2^ · s^−1^ (*t*_4_ = −0.17, *p* = 0.877), 6 μmol photons · m^−2^ · s^−1^ (*t*_3_ = −1.73, *p* = 0.192), 15 μmol photons · m^−2^ · s^−1^ (*t*_4_ = −0.63, *p* = 0.568), 56 μmol photons · m^−2^ · s^−1^ (*t*_4_ = −2.65, *p* = 0.058), 170 μmol photons · m^−2^ · s^−1^ (*t*_3_ = −1.18, *p* = 0.326), 242 μmol photons · m^−2^ · s^−1^ (*t*_3_ = −1.27, *p* = 0.290), and 348 μmol photons · m^−2^ · s^−1^ (*t*_4_ = 1.52, *p* = 0.205). However, substantial differences were observed at 24 μmol photons · m^−2^ · s^−1^ (*t*_4_ = −4.54, *p* = 0.011), 90 μmol photons · m^−2^ · s^−1^ (*t*_3_ = −3.09, *p* = 0.048), and 120 μmol photons · m^−2^ · s^−1^ (*t*_4_ = −5.99, *p* = 0.005).

**FIGURE 6:**
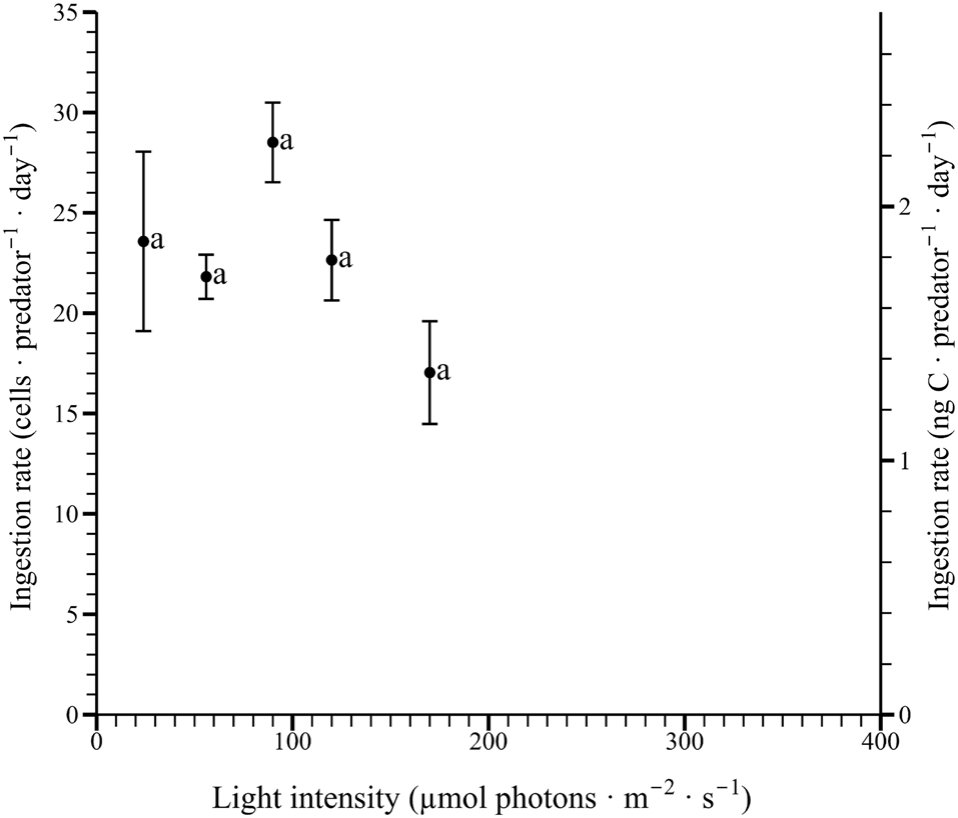
Ingestion rates of *Pyrophacus horologium* under different light intensities. The light intensities of 0, 6, 15, 242, and 348 μmol photons · m^−2^ · s^−1^ were excluded due to negative growth rates. Each point represents the mean of three replicates, and vertical error bars indicate ±1 standard error. According to Tukey's HSD test, the groupings for ingestion rates were as follows: 24–170 μmol photons · m^−2^ · s^−1^ (a).

In mixotrophic conditions, the ingestion rates of *Pyrophacus horologium* were measured across a light intensity range of 24–170 μmol photons · m^−2^ · s^−1^, ranging from 17.0–28.5 cells · predator^−1^ · day^−1^ (Figure 6). The 0, 6, 15, 242, and 348 μmol photons · m^−2^ · s^−1^ conditions, where negative growth rates were observed, were excluded from the ingestion rate analysis. The maximum ingestion rate was recorded at 90 μmol photons · m^−2^ · s^−1^. The results of one‐way ANOVA revealed that the ingestion rate did not differ significantly depending on light intensity (*F*_4,10_ = 2.36, *p* = 0.124), and no significant groupings were identified through Tukey's HSD post hoc test (*p* > 0.05; Figure 6). One‐tailed *t*‐tests conducted for each light intensity condition indicated that ingestion rates were significantly higher than zero at all tested light intensities. The following light intensities were identified as significant: 24 μmol photons · m^−2^ · s^−1^ (*t*_2_ = 5.28, *p* = 0.017), 56 μmol photons · m^−2^ · s^−1^ (*t*_2_ = 19.9, *p* = 0.001), 90 μmol photons · m^−2^ · s^−1^ (*t*_2_ = 14.3, *p* = 0.002), 120 μmol photons · m^−2^ · s^−1^ (*t*_2_ = 11.3, *p* = 0.004), and 170 μmol photons · m^−2^ · s^−1^ (*t*_2_ = 6.66, *p* = 0.011).

### Swimming speed

The mean swimming speeds of *Pyrophacus horologium* and *Hetercapsa. niei* were measured to be 274 μm · s^−1^ (*n* = 35, ± 3.14 *SE*) and 413 μm · s^−1^ (*n* = 35, ± 6.54 *SE*), respectively. The maximum recorded swimming speeds were found to be 320 μm · s^−1^ and 497 μm · s^−1^, respectively.

## DISCUSSION

### Prey concentration effects

Under autotrophic conditions, the growth of *Pyrophacus horologium* was considerably lower than under mixotrophic conditions, indicating that prey availability strongly influences its overall growth performance. However, as predator density increased, growth rates decreased to approximately 1/3–1/5 of the original level. Although not definitive, this decrease may be attributable to suboptimal environmental conditions, as the experiment was conducted prior to the temperature and irradiance trials at 22°C and 90 μmol photons · m^−2^ · s^−1^. The irradiance levels were within the optimal range (IOGZ: 68.13–115.79 μmol photons · m^−2^ · s^−1^), whereas the temperature was below the optimal range (TOGZ: 28.3–31.0°C). Consequently, the relatively low growth rates observed may have been driven, at least in part, by these conditions. Given the relatively low cell densities and the use of F/2‐Si medium, nutrient limitation is unlikely to have occurred. Therefore, given the prevailing uncertainty, the observed decline in growth rate may be interpreted as resulting from transient physiological stress or environmental suboptimality.

Under mixotrophic conditions, growth was approximately twofold higher than under autotrophic conditions, presumably due to a sufficient prey supply (Figure 1). However, the results also demonstrated that mixotrophic growth was inhibited as prey density increased. As in the autotrophic condition, this pattern may, at least in part, reflect disrupted conditions relative to the growth optimum of the species. In addition, elevated prey concentrations may have rapidly deteriorated culture water quality. As Kim et al. (2008) reported, in mixed cultures of the predator *Dinophysis acuminata* and its prey *Mesodinium rubrum*, growth was increasingly inhibited at higher prey densities, and they suggested that a rapid increase in pH driven by dense prey could suppress predator growth. Furthermore, Jeong and Latz (1994) proposed that bloom‐forming prey species, when present at high densities, may have a detrimental effect on predator proliferation through prey‐derived toxicity and/or physical interference. Collectively, these mechanisms may account for the post‐saturation downturn observed in the growth curve. Conversely, no inhibitory effects were detected in subsequent assays conducted under near‐optimal temperature and irradiance conditions, suggesting that such inhibition may be mitigated under more favorable conditions. Nevertheless, given that toxicity was not directly measured in this study, targeted verification is required. It is noteworthy that when autotrophic and mixotrophic conditions were compared at similar predator densities, the decline in growth observed under autotrophy was not evident under mixotrophy, where growth remained relatively stable. The results demonstrate that *Pyrophacus horologium* can maintain favorable growth even under unfavorable conditions via a mixotrophic strategy. Consequently, evaluations of the growth characteristics and ecological functions of this species should explicitly consider its mixotrophic capabilities.

*Pyrophacus horologium* is a widely distributed species, with reported occurrences in various regions, including the southern coast of China (Gul et al., 2018; Nunes et al., 2019; Seong et al., 2025; Yan et al., 2002). The southern waters of China are home to the toxic dinoflagellate *Hetercapsa niei*, which causes harmful algal blooms (HABs) and has been reported to cause ecological damage such as shellfish mortality (Wu et al., 2022). *Pyrophacus horologium* has been identified as the first predator to consume *H. niei*, and it is noteworthy that it can coexist with toxic species without being affected by allelopathy or toxicity. These characteristics suggest that *P. horologium* may function as a natural regulator in waters dominated by *H. niei*, indicating its potential for high removal rates. Notably, field HABs densities (ca. 3500 cells · mL^−1^) are lower than the upper prey level used in our experiments (ca. 24,000 cells · mL^−1^), indicating that clearance‐based removal derived in this study is likely to operate effectively within this mid‐density range. However, the realized mitigation efficiency will ultimately depend on the initial density of *P. horologium*. Consequently, numerical modeling and mesocosm‐based studies are necessary to quantitatively assess the population dynamics of *P. horologium* and the control efficiency of *H. niei* in the future.

The genus *Heterocapsa* is ubiquitous in the world's oceans, with certain species inducing HABs and concurrently serving as a primary sustenance source for diverse predators (Oda et al., 2001; Saburova et al., 2022; Stoecker & Sanders, 1985). Consequently, previous studies on predators that feed on *Heterocapsa* species were referenced to enhance understanding of interspecific comparisons in this study. As demonstrated in Table 2, the maximum swimming speed of the predator *Aduncodinium glandula* has been recorded to be 546 μm · s^−1^. In contrast, the maximum swimming speeds of its prey, *H. triquetra* and *H. rotundata*, have been reported to be 496 and 398 μm · s^−1^, respectively (Jeong et al., 2002; Skovgaard & Hansen, 2003). In such cases, predators have been observed to exhibit swimming speeds that exceed those of their prey. In contrast, the maximum swimming speed of *Pyrophacus horologium* was comparatively low at 320 μm · s^−1^, whereas *H. niei* exhibited a faster swimming speed of 497 μm · s^−1^. However, *P. horologium* exhibited an *I*_max_ of 28.0 cells · predator^−1^ · day^−1^ (Figure 2), indicating that feeding occurred through instantaneous predatory behavior or allelopathic effects exerted by *P. horologium*. The allelopathic activity of *P. horologium* has been reported to induce partial cell death and reduced motility in *H. niei* (Seong et al., 2025). The findings indicate that *P. horologium* possesses the ecological potential to function as an effective predator in the marine environment, despite its relatively limited swimming ability. Concurrently, the mixotrophic dinoflagellate *Gymnodinium smaydae* exhibited a markedly high μ_max_ (2.23 · day^−1^) when feeding on *H. rotundata*, whereas the μ_max_ of *P. horologium* was relatively lower (0.20 · day^−1^). Nevertheless, the *I*_max_ of *P. horologium* (2.21 ng C · predator^−1^ · day^−1^) exhibited a higher value than that of *G. smaydae* (1.59 ng C · predator^−1^ · day^−1^). In comparison to the ciliate *Tiarina fusus*, *P. horologium* exhibited a slightly lower *I*_max_ (2.21 ng C · predator^−1^ · day^−1^), but the values were considered comparable (2.60 ng C · predator^−1^ · day^−1^ for *T. fusus*). In addition, when compared to the majority of other predators except *Gyrodinium spirale*, *P. horologium* showed an *I*_max_ close to the mean value of 1.89 ng C · predator^−1^ · day^−1^ (Table 2). These results suggest that *P. horologium* has a feeding capacity comparable to that reported for most documented *Heterocapsa* predators, excluding *Gr*. *spirale*, under the tested conditions.

**TABLE 2:** The maximum mixotrophic growth rates (μ_max_) and maximum ingestion rates (*I*_max_) of various protistan predators feeding on *Heterocapsa* spp. were estimated using the Michaelis–Menten equation.

Predator	ESD	MSS	Prey	ESD	μ_max_	*I*_max_	Tm	Li	References
*Aduncodinium glandula* (HTD)	21.0	546	*Heterocapsa triquetra* (MTD)	15.0	1.00	0.75	20	20	Jang et al. (2016)
*Gymnodinium smaydae* (MTD)	10.6	707	*Heterocapsa rotundata* (MTD)	9.5	2.23	1.59	20	20	Lee et al. (2014)
*Gyrodinium dominans* (HTD)	20.0	‐	*Heterocapsa triquetra* (MTD)	15.3	0.54	2.30	20	100	Nakamura et al. (1995)
*Gyrodinium spirale* (HTD)	31.8	‐	*Heterocapsa triquetra* (MTD)	15.8	1.08	7.50	15	5	Hansen (1992)
*Pyrophacus horologium* (MTD)	49.4	320	*Heterocapsa niei*	10.1	0.20	2.21	22	90	This study
*Tiarina fusus* (HTC)	37.0	3125	*Heterocapsa triquetra* (MTD)	12.8	−0.08	2.60	19	10	Jeong et al. (2002)

Abbreviations: ESD, equivalent spherical diameter (μm); HTC, heterotrophic ciliate; HTD, heterotrophic dinoflagellate; Li, light intensity (μmol photons · m^−2^ · s^−1^); MSS, maximum swimming speed (μm · s^−1^) μ_max_ (day^−1^); *I*_max_ (ng C · predator^−1^ · day^−1^); MTD, mixotrophic dinoflagellate; Tm, temperature (°C).

### Temperature effects

Across the temperature treatments, the control growth rates of *Heterocapsa niei* and *Pyrophacus horologium* exhibited similar optimal responses between 20 and 32°C (Figures 3 and S3). The observed temperature range was conducive to the growth of both species; mixotrophic conditions within this range are expected to support enhanced net growth of the predator through the concurrent fulfillment of sufficient prey availability and active feeding. However, the tendency for the maximum ingestion rate to occur at a slightly lower temperature than the peak growth rate can be explained by a slight decoupling arising from differing temperature dependencies of feeding efficiency and assimilation, repair/photosynthetic efficiency (Flynn & Mitra, 2009; Menden‐Deuer & Grünbaum, 2006).

The majority of mixotrophic dinoflagellates exhibited optimal growth temperatures of approximately 25°C, as indicated in Table 3. In contrast, *Pyrophacus horologium* exhibited optimal growth rates at 30°C, suggesting physiological characteristics analogous to those observed in dinoflagellates inhabiting tropical and subtropical seas. Specifically, *P. horologium* has an equivalent spherical diameter (ESD) of 49.4 μm, compared to 32.0 μm for *Alexandrium pohangense*, as reported by Lim et al. (2019). Given the observed size difference, the maximum ingestion rate recorded by *P. horologium* at 28°C (2.39 ng C · predator^−1^ · day^−1^) can be evaluated as similar to that of *A. pohangense* in terms of ingestion efficiency for the harmful dinoflagellate *Heterocapsa niei*. The elevated growth rate exhibited under mixotrophic conditions suggests that energy acquisition through feeding is parallel to photosynthesis, reflecting a physiological strategy similar to that of *A. pohangense*, which shows superiority in high‐temperature environments through a mixotrophic strategy. These results suggest that *P. horologium* may occupy a dominant position in the ecosystem in an environment with rising sea temperatures.

**TABLE 3:** The maximum growth and ingestion rates, and optimal physiological conditions of various mixotrophic dinoflagellates under different temperature and light intensity conditions.

Species	Optimal prey	OT	MMGT	MIT	MAGT	OLI	MMGL	MIL	MAGL	References
*Alexandrium pohangense*	*Margalefdinium polykrikoides* (MTD)	30	0.64	3.50 (32)	0.08 (20–25)	25	0.28	1.90 (58)	0.17	Lim et al. (2019)
*Biecheleria* *cincta*	*Heterosigma* *akashiwo* (MTR)	25	0.26	0.40	−0.09 (15)	‐	‐	‐	‐	You, Jeong, et al. (2023)
*Dinophysis acuminata*	*Mesodinium* *rubrum* (MTC)	24	0.61	‐	‐	100	0.61	‐	‐	Fiorendino et al. (2020)
*Dinophysis* *ovum*	*Mesodinium* *rubrum* (MTC)	24	0.47	‐	‐	50	0.47	‐	‐	Fiorendino et al. (2020)
*Fragilidium subglobosum*	*Ceratium tripos* (MTD)	‐	‐	‐	‐	45	0.5	7.32	0.16	Hansen and Nielsen (1997), Hansen et al. (2000)
*Gymnodinium smaydae*	*Heterocapsa rotundata* (MTD)	25	1.55	4.20 (32)	−0.05 (20)	58	1.28	2.30	−0.12 (0)	You et al. (2020)
*Paragymnodinium shiwhaense*	*Amphidinium carterae* (MTD)	25	1.16	0.46 (20)	‐	100	1.17	0.42 (10)	‐	Jeong et al. (2018)
*Pyrophacus horologium*	*Heterocapsa* *niei*	30	0.48	2.39 (28)	0.25	120	0.38	2.25 (90)	0.11	This study
*Takayama helix*	*Alexandrium minutum* (MTD)	26	0.42	0.95 (15)	0.23 (28)	15	0.42	2.00 (350)	0.20	Ok et al. (2019)
*Yihiella* *yeosuensis*	*Teleaulax amphioxeia* (MTC)	25	1.16	0.24	0.16 (15)	‐	‐	‐	‐	Kang et al. (2020)

*Note*: All predators are classified as mixotrophic dinoflagellates. The values in parentheses indicate that the rates were not obtained under optimal conditions and represent the observed values at the corresponding conditions.

Abbreviations: MAGL, maximum autotrophic growth rate in the light intensity experiments (day^−1^); MAGT, maximum autotrophic growth rate in the temperature experiments (day^−1^); MIL, maximum ingestion rate in the light intensity experiments (ng C · predator^−1^ · day^−1^); MIT, maximum ingestion rate in the temperature experiments (ng C · predator^−1^ · day^−1^); MMGL, maximum mixotrophic growth rate in the light intensity experiments (day^−1^); MMGT, maximum mixotrophic growth rate in the temperature experiments (day^−1^); MTC, mixotrophic cryptophyte; MTCI, mixotrophic ciliate; MTD, mixotrophic dinoflagellate; MTR, mixotrophic rhaphidophyte; OLI, optimal light intensity (μmol photons · m^−2^ · s^−1^); OT, optimal temperature (°C).

**FIGURE 7:**
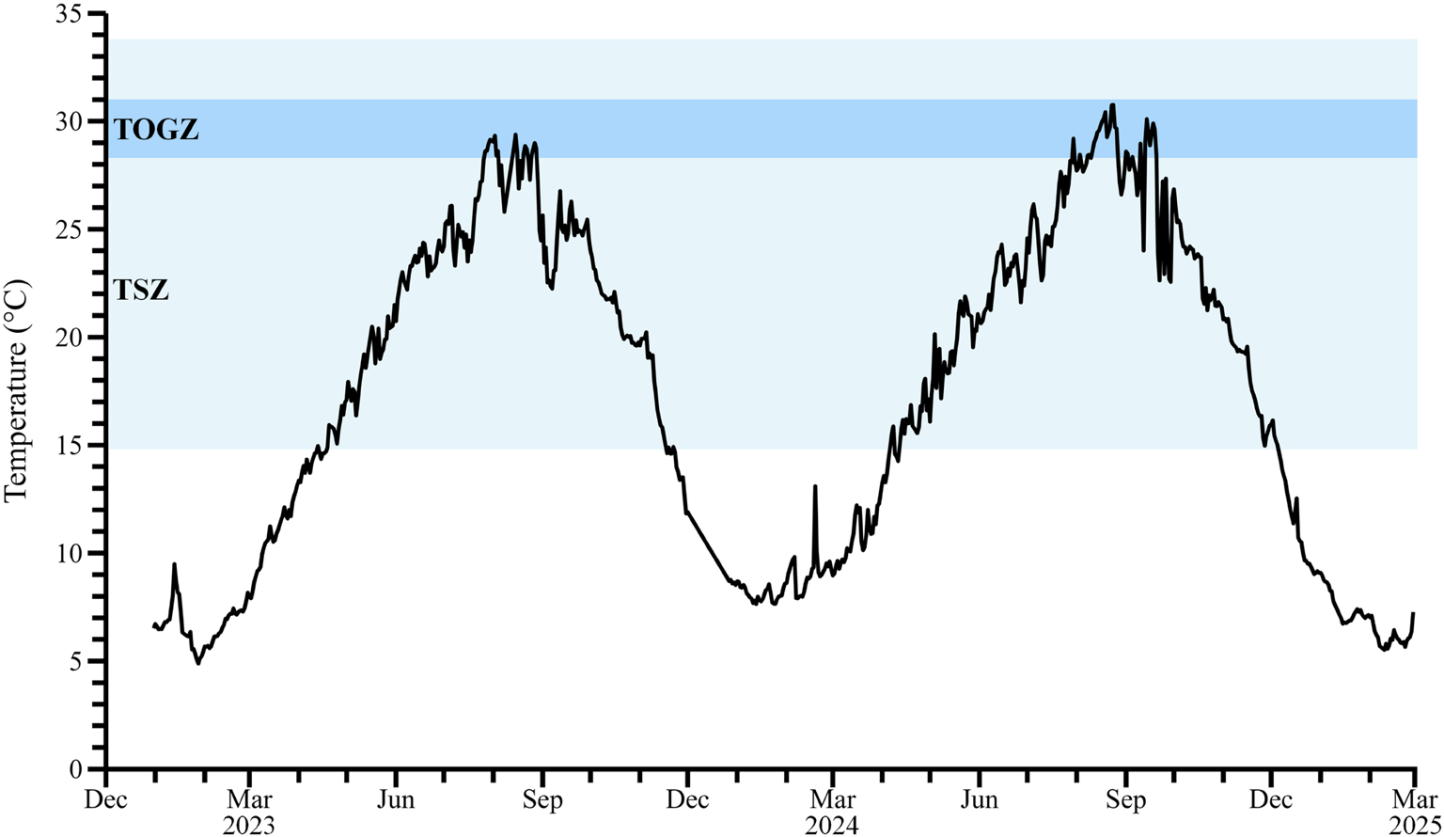
Daily mean water temperature time series at the isolation site of *Pyrophacus horologium* from January 2023 to February 2025, with temperature ranges defined using spline functions. Based on the measured temperature data, the temperature‐based survival zone (TSZ; 14.8–33.8°C) and temperature‐based optimal growth zone (TOGZ; 28.3–31.0°C) were determined.

Within the temperature‐based survival zone (TSZ; 14.8–33.8°C) and the temperature‐based optimal growth zone (TOGZ; 28.3–31.0°C) identified in Figure 7, the TOGZ was primarily observed between July and September, whereas the TSZ extended from April to November. This pattern indicates that *Pyrophacus horologium* can maintain positive growth across a broad thermal range, with optimal proliferation occurring during the summer period. In contrast, *P. horologium* has been observed to form resting cysts, which facilitate survival under conditions that are suboptimal, such as low temperatures or nutrient depletion (Matsuoka, 1985). This encystment ability is hypothesized to enable the species to persist outside the TSZ in field observations and subsequently germinate when conditions become favorable once more (Figures 3 and 7).

The present study hypothesizes that *Pyrophacus horologium* may gain an ecological advantage over competing species through rapid growth and high feeding rates within the TOGZ. Concurrently, Han et al. (2023) reported that the sea surface temperature along the coast of the Korean Peninsula has increased at a rate approximately 2.6 times faster than the global average over the past 55 years. This long‐term warming trend suggests that the duration of the period corresponding to the TOGZ will gradually extend over the coming decades to centuries. Consequently, during periods of overlap between sea surface temperatures and the TOGZ, the environmental conditions conducive to the dominance of *P. horologium* may expand in concert. Consequently, *P. horologium*, a mixotrophic dinoflagellate inhabiting high‐temperature environments, can serve as a meaningful indicator organism for understanding changes in ecosystem structure and function under future climate change scenarios.

The species *Pyrophacus horologium* exhibited stable growth even under elevated temperature conditions, demonstrating a pronounced tolerance to high temperatures. This physiological trait differentiates it from other mixotrophic dinoflagellates and suggests that it may act as a potential dominant species in future marine environments characterized by sustained ocean warming. Furthermore, *P. horologium* has been observed to utilize prey species that are typically not consumed by other mixotrophic dinoflagellates, including members of the genera *Gonyaulax* and *Alexandrium*, as well as *Heterocapsa* (Seong et al., 2025). The broad spectrum of prey highlights the suitability of this species as a model organism for investigating mixotrophy within dinoflagellate food webs. Nevertheless, future studies should aim to quantify the relative contributions of photosynthesis and phagotrophy and, as demonstrated in previous works (e.g., Hansen, 2011; Skovgaard, 1996), assess changes in photosynthetic efficiency under varying prey availability and light conditions to further advance our understanding of *P. horologium*. This approach will contribute to understanding the adaptation of *P. horologium* to mixotrophic conditions in future marine environments.

### Light effects

*Heterocapsa niei* (control) exhibited marked growth inhibition under high irradiance (170–348 μmol photons · m^−2^ · s^−1^). Net growth became negative at irradiances of 170 μmol photons · m^−2^ · s^−1^ and above. However, at 242 μmol photons · m^−2^ · s^−1^, this inhibition was transiently alleviated. This response may be consistent with photosystem II repair and photoprotective processes (e.g., xanthophyll cycling and antenna regulation) as was described during acclimation in previous studies (Aro et al., 1993; Ruban, 2016; Han et al., 2000). In contrast, growth inhibition was more pronounced at 170 and 348 μmol photons · m^−2^ · s^−1^ than at 242 μmol photons · m^−2^ · s^−1^. This pattern may be consistent with photoprotective responses being absent or insufficient, or with protective and repair capacities being exceeded, thereby allowing chronic photoinhibition to predominate (Aro et al., 1993; Eilers & Peeters, 1988; Ruban, 2016). Nevertheless, because photosystem II performance and photoprotective pigments were not directly measured in the present study, the proposed mechanisms are presented as literature‐based interpretations and require targeted verification.

Under various temperature conditions, the contribution of the mixotrophic type was relatively low; however, under various light intensity conditions, the growth‐promoting effect was more pronounced. Within the range of 24–170 μmol photons · m^−2^ · s^−1^, a discernible disparity in growth rate was evident between the two trophic types. This phenomenon is analogous to the results of *Fragilidium subglobosum* and *Takayama helix*, whose growth rate and ingestion rate vary greatly depending on light intensity (Ok et al., 2019; Skovgaard, 1996). Conversely, under conditions of low light (0 μmol photons · m^−2^ · s^−1^), both autotrophic and mixotrophic growth rates were negative, and *Pyrophacus horologium* was classified as a Darkness‐Type I mixotrophic dinoflagellate according to the classification criteria of Ok et al. (2019). According to this classification, Darkness‐Type I species cannot grow in complete darkness even when prey is available, whereas Darkness‐Type II species are capable of sustaining growth by utilizing prey as an energy source. *Pyrophacus horologium* exhibited characteristics similar to those of *Paragymnodinium shiwhaense* and *T. helix*, which are also classified as Darkness‐Type I, reflecting the characteristics of species with limited survival or feeding abilities under dark conditions (Jeong et al., 2018; Ok et al., 2019). Despite being provided with *Heterocapsa niei* as prey, *P. horologium* did not exhibit feeding activity under dark conditions, and its ingestion rate was not detected, indicating that it could not sustain growth. In contrast, the phylogenetically related species *F. subglobosum* is classified as Darkness‐Type II and was able to maintain positive growth under dark conditions when prey was available (Skovgaard, 1996). This finding suggests that species with similar phylogenetic origins may possess different strategies for carbon acquisition.

The feeding rate at different light levels indicates that *Pyrophacus horologium* possesses the ability to maintain growth under specific light conditions. Nevertheless, reduced light availability or complete darkness may impede feeding induction or prey capture efficiency. At an irradiance of 120 μmol photons · m^−2^ · s^−1^, the irradiance at which the maximum growth rate was achieved, the ingestion rate was relatively low. This result suggests that photosynthetic carbon acquisition was highly efficient under this light intensity, leading to a reduced dependence on heterotrophic feeding. *Pyrophacus horologium* employs a strategy of optimizing survival and growth by performing vertical movement to avoid low or excessive light conditions. This phenomenon is analogous to the diel vertical migration pattern commonly observed in dinoflagellates, characterized by upward movement during the day and downward movement at night (Ji & Franks, 2007; Ralston et al., 2007). Consequently, further investigation into the vertical distribution of *P. horologium*, taking into account its interactions with the key prey organism *Heterocapsa niei*, is warranted.

**FIGURE 8:**
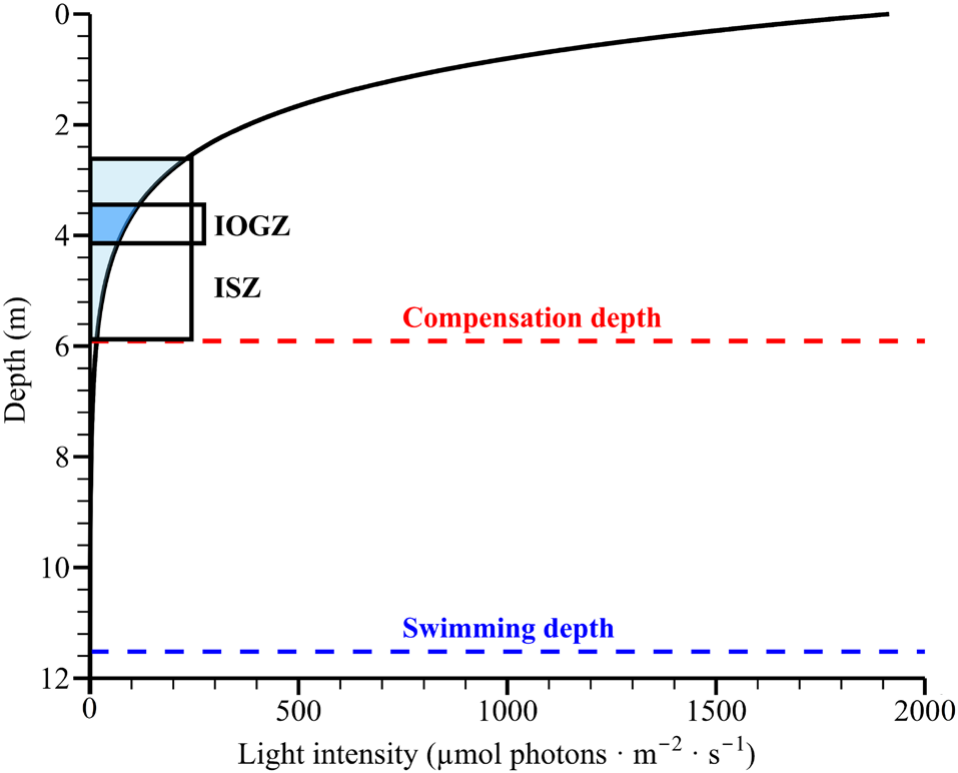
Spline‐based depth and irradiance range settings related to the growth of *Pyrophacus horologium*. Based on the attenuation of photosynthetically active radiation (PAR) with depth, the irradiance‐based survival zone (ISZ; 16.06–233.26 μmol photons · m^−2^ · s^−1^, 2.60–5.91 m) and irradiance‐based optimal growth zone (IOGZ; 68.13–115.79 μmol photons · m^−2^ · s^−1^, 3.47–4.12 m) were defined. In addition, the compensation depth (16.02 μmol photons · m^−2^ · s^−1^, 5.91 m) and the maximum swimming depth (0.17 μmol photons · m^−2^ · s^−1^, 11.52 m) are indicated as reference baselines.

The irradiance‐based optimal growth zone (IOGZ; 3.47–4.12 m) and irradiance‐based survival zone (ISZ; 2.60–5.91 m) were estimated in conjunction with August PAR data and the mean Secchi disk transparency observed in Masan Bay (Figure 8). A consideration of the observed minimum and maximum values of water transparency reveals that the depth range at which the maximum growth light intensity (120 μmol photons · m^−2^ · s^−1^) could be reached extends from 2.77 to 4.89 m. This range indicates the optimal light environment in which *Pyrophacus horologium* can effectively reside through diel vertical migration. Furthermore, given the observed swimming speed, the depth range shallower than the theoretically reachable maximum depth (11.52 m) can be interpreted as the effective depth zone where *P. horologium* can remain for growth and feeding.

*Pyrophacus horologium* and *Heterocapsa niei* exhibited similar growth patterns across the irradiance gradient. Maximum growth rates were observed for both species at 120 μmol photons · m^−2^ · s^−1^, and light responses were similar across the shared range of positive growth (24–120 μmol photons · m^−2^ · s^−1^). These results suggest that, in natural marine environments when the distributions of the two species overlap, interactions may occur under the same light conditions. These light‐dependent growth characteristics may serve as useful biological parameters in future studies involving biomass estimation and numerical modeling.

## CONCLUSIONS

*Pyrophacus horologium* was quantitatively evaluated for its growth and ingestion rates under varying prey concentrations, temperatures, and light intensities while feeding on the harmful dinoflagellate *Heterocapsa niei*. *Pyrophacus horologium* demonstrated robust growth in conditions of relatively high temperature. The light intensity exhibited a significant effect on growth, with optimal growth rates observed at 120 μmol photons · m^−2^ · s^−1^ (at 30°C). In the majority of experimental conditions, the growth rate of the mixotrophic group exceeded that of the autotrophic group. In conditions of darkness, the growth of *P. horologium* ceased, and no ingestion occurred. Consequently, *P. horologium* is classified as a Darkness‐Type I mixotrophic dinoflagellate. Furthermore, despite swimming at slower speeds than their prey, high ingestion rates were recorded, suggesting the possibility of feeding through instantaneous predation mechanisms or allelopathic effects. Although these results underscore the ecological potential of *P. horologium* as an effective predator of the harmful dinoflagellate *H. niei* and as a climate change responsive species that may secure a competitive advantage in warming coastal ecosystems, they were derived under controlled laboratory conditions. In addition, the effects of salinity were not a primary focus of this study. It is therefore recommended that future research quantify the growth and ingestion responses and delineate tolerance ranges of *P. horologium* to climate‐linked hyposalinity and hypersalinity events (e.g., storm‐driven freshening). Further studies should assess how environmental variability, such as nutrient availability, small‐scale turbulence, and competitive interactions with other protists, influences the species' growth and feeding behaviors in natural settings. Such studies should also characterize interactions across a broader prey spectrum to establish the trophic position of *P. horologium* within natural planktonic food webs. Collectively, these endeavors will furnish a more comprehensive understanding of its population dynamics and the potential ramifications for the stability of coastal ecosystems.

## AUTHOR CONTRIBUTIONS

**Min‐jun Seong:** Conceptualization (equal); data curation (equal); formal analysis (equal); investigation (equal); methodology (equal); software (equal); writing – original draft (equal). **Kun‐woo Yun:** Data curation (equal); formal analysis (equal); methodology (equal); project administration (equal); validation (equal); writing – review and editing (equal). **Hwa‐seong Son:** Conceptualization (equal); formal analysis (equal); investigation (equal); methodology (equal); validation (equal). **Seung‐min Lee:** Formal analysis (equal); investigation (equal); validation (equal). **Mu‐chan Kim:** Conceptualization (lead); data curation (lead); funding acquisition (equal); methodology (equal); project administration (lead); resources (lead); supervision (lead); validation (lead); writing – original draft (supporting); writing – review and editing (equal).

## Supporting information


**Figure S1.** Acclimation and experimental temperature schemes for each target temperature.
**Figure S2.** Acclimation and experimental phases for each target light intensity.
**Figure S3.** Autotrophic growth rates of *Heterocapsa niei* (prey‐only control) under different temperature conditions. Each point represents the mean of three replicates, and vertical error bars indicate ±1 standard error.
**Figure S4.** Autotrophic growth rates of *Heterocapsa niei* (prey‐only control) under different light intensities. Each point represents the mean of three replicates, and vertical error bars indicate ±1 standard error.
