# Supplementary material for: Influence of prey concentration, light intensity, and temperature on the growth and ingestion of the mixotrophic dinoflagellate Pyrophacus horologium, a predator of the harmful species Heterocapsa niei
Source: J Phycol. 2026 Mar 16;62(2):606–22. doi: 10.1111/jpy.70150 (PMC13103703; doi:10.1111/jpy.70150)
Supplement: Supplementary file 1 — Figure S1. Acclimation and experimental temperature schemes for each target temperature. Figure S2. Acclimation and experimental phases for each target light intensity. Figure S3. Autotrophic growth rates of Heterocapsa niei (prey‐only control) under different temperature conditions. Each point represents the mean of three replicates, and vertical error bars indicate ±1 standard error. Figure S4. Autotrophic growth rates of Heterocapsa niei (prey‐only control) under different light intensities. Each point represents the mean of three replicates, and vertical error bars indicate ±1 standard error. [file JPY-62-606-s001.docx]

Tm: Temperature

**Figure S1.** Acclimation and experimental temperature schemes for each target temperature.

Li: light intensity

**Figure S2.** Acclimation and experimental phases for each target light intensity.


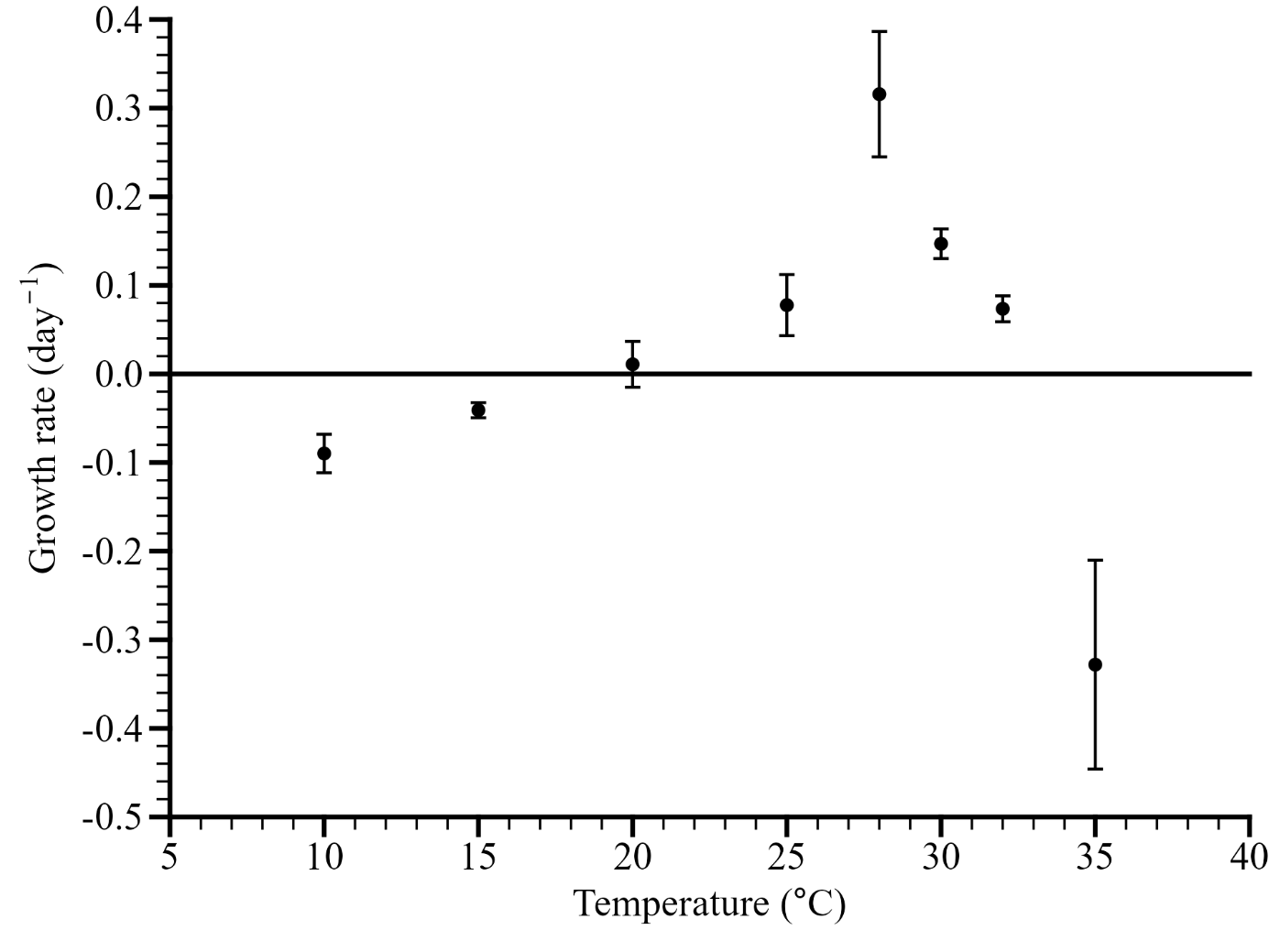


**Figure. S3** Autotrophic growth rates of *Heterocapsa niei* (prey-only control) under different temperature conditions. Each point represents the mean of three replicates, and vertical error bars indicate ±1 standard error.


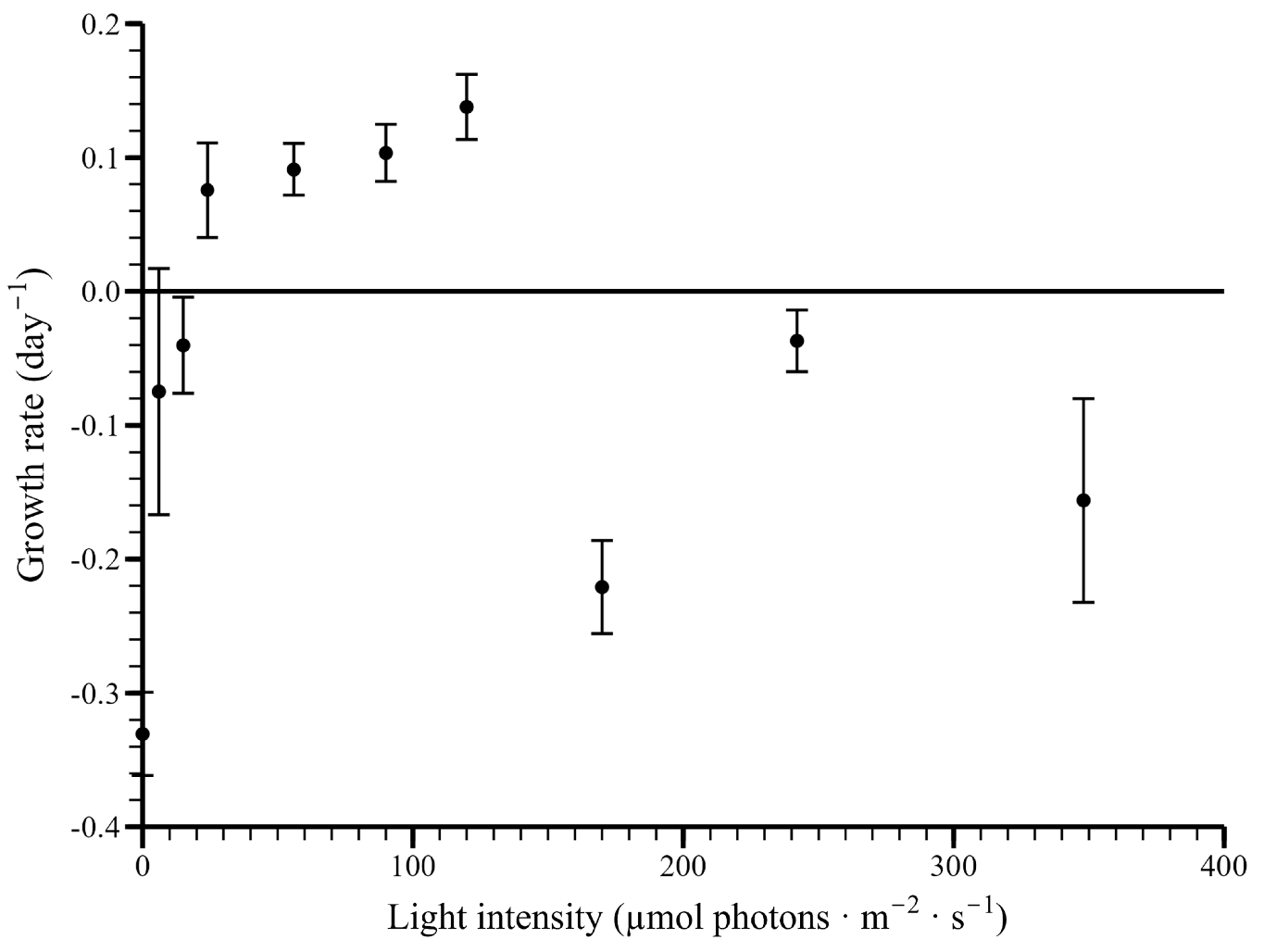


**Figure. S4** Autotrophic growth rates of *Heterocapsa niei* (prey-only control) under different light intensities. Each point represents the mean of three replicates, and vertical error bars indicate ±1 standard error.
